# Characterization and phylogenetic analysis of the complete mitochondrial genome of *Actinopyga lecanora* (Jaeger, 1833) (Holothuriida: Holothuriidae)

**DOI:** 10.1080/23802359.2021.1970641

**Published:** 2021-08-31

**Authors:** Shengping Zhong, Ying Qiao, Longyan Zhao, Guoqiang Huang, Yonghong Liu, Lianghua Huang

**Affiliations:** a Institute of marine drugs, Guangxi University of Chinese Medicine, Nanning, China; b Guangxi Engineering Technology Research Center for Marine Aquaculture, Guangxi Institute of Oceanology Co., Ltd, Beihai, China; c Ministry of Natural Resources, Fourth institute of Oceanography, Beihai, China

**Keywords:** Mitochondrial genome, *Actinopyga lecanora*, cryptic species

## Abstract

The white-bottomed sea cucumber, *Actinopyga lecanora*, is a valuable inshore fisheries resource which is famous for its high nutrition and pharmacological compounds. However, due to morphological plasticity and limited molecular phylogenetic studies, the taxonomic histories in the genus *Actinopyga* have not been completely resolved yet. Moreover, there remains a complex of cryptic species within *Actinopyga,* many of which are incorrectly assigned within the family Holothuriidae. In this study, we report the complete mitochondrial genome of *A. lecanora*. The mitogenome has 15,568 base pairs (63.40% A + T content) and is made up of a total of 37 genes (13 protein-coding, 22 transfer RNAs and 2 ribosomal RNAs), plus a putative control region. This study offers useful mitogenome data for future phylogenetic and taxonomic classification of Holothuriidae.

Sea cucumbers (Holothuroidea), commonly known as holothuroids, are a species-rich group of relatively large benthic invertebrates which only inhabit in marine environments (Miller et al. 2017). Many sea cucumber species play an important role in marine benthic ecosystem due to their huge abundance in local benthic community (Uthicke 2001). Moreover, holothuroids are important coastal fisheries resources, many of which have highly commercial value in the Southeast Asian market especially in China (Anderson et al. 2011). *Actinopyga lecanora* is a common edible sea cucumber widely distributed throughout the tropical Indo-west Pacific region and has been known as functional food for its relatively high protein and anti-oxidative component (Ghanbari et al. 2015). Despite its abundance and economically importance, many sea cucumbers in the genus *Actinopyga* are being commercialized without clearly taxonomic identification (Ahmed 2016) and the mitogenomes of all species in the genus *Actinopyga* have yet to be sequenced. Mitogenome sequences have proven to be excellent molecular resources for analyzing evolutionary and phylogenetic relationship (Madduppa et al. 2017). Here, we report the complete mitochondrial genome of *A. lecanora*, which will afford useful molecular data for taxonomic and phylogenetic analyses in sea cucumbers.

One individual sample of *A. lecanora* was collected from the HaiNan province, China (LingShui, 18.384207 N, 109.981511 E) by local diving fishermen. And the whole body specimen (#GH0078) was deposited at Marine biological Museum, Guangxi Institute of Oceanology, Beihai, China (http://www.gxas.cn/kypt/kxpj/kpcg, Shengping Zhong, shpzhong@foxmail.com). The total genomic DNA was extracted from the muscle of the specimen using an SQ Tissue DNA Kit (OMEGA, Guangzhou, China) following the manufacturer’s protocol. DNA libraries (350 bp insert) were constructed with the TruSeq NanoTM kit (Illumina, San Diego, CA) and were sequenced (2 × 150 bp paired-end) using HiSeq platform at BGI Company, China. Mitogenome assembly was performed with MITObim (Hahn et al. 2013). The complete mitogenome sequence of *A. echinites* (GenBank accession number: MN793975) (Zhong et al. 2020) was chosen as the initial reference sequence for MITObim assembly. Gene annotation was performed by MITOS (Bernt et al. 2013).

The complete mitogenome of *A. lecanora* (GenBank accession number: MW248463) is 15,568 bp in length and it contains a conserved set of 13 protein-coding genes (PCGs), 2 ribosomal RNA genes, 22 transfer RNA genes, and a putative control region. A total of 37 genes were annotated and 227 nucleotides were identified as putative control region. The overall base composition of the mitogenome is estimated to be A 34.64%, T 28.76%, C 21.47% and G 15.13%, with a high A + T content of 63.40%, which is similar, but slight higher than *A. echinites* (62.94%) (Zhong et al. 2020). The phylogenetic analysis inferred from the concatenated nucleotides sequences of 13 PCGs suggests that *Actinopyga* and *Holothuria* holothuroids have closely relationship in family Holothuriidae (Figure 1), which is consistent with the phylogenetic analyses of holothuroids from Indonesia using mitochondrial Cytochrome c oxidase 1 (COI) sequences (Madduppa et al. 2017). The mitogenome of *A. lecanora* identified in this study exhibited 99.52% identity to the “hui” strain (GenBank accession number: MW218894) indicates that *A. lecanora* have very closely genetic relationship between these strains. These findings will be helpful for better resolving its taxonomic controversy and future conservation.

**Figure 1.**
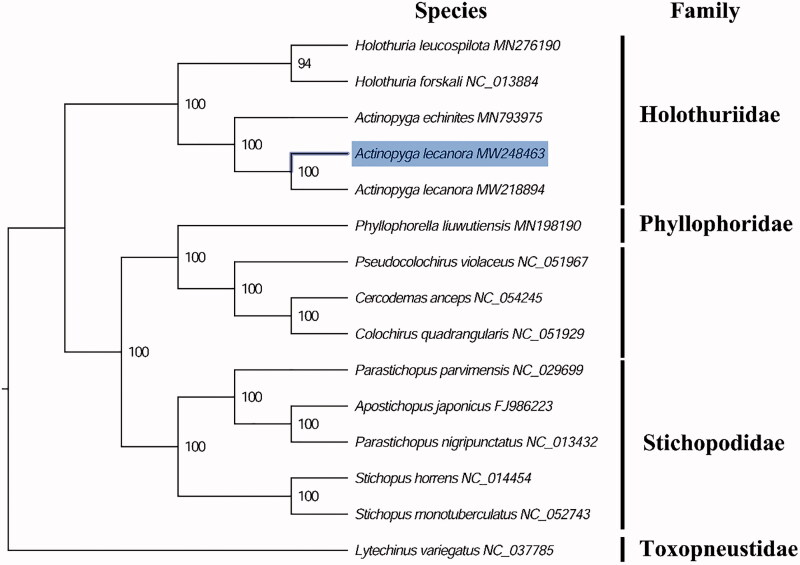
Phylogenetic tree of 15 species in echinoderms. The complete mitogenomes were downloaded from GenBank and the phylogenic tree based on the concatenated nucleotide sequences of 13 mitochondrial PCGs was constructed by maximum-likelihood method via PhyML online server (http://www.atgc-montpellier.fr/phyml/), using GTR substitution model with 100 bootstrap replicates. The bootstrap values are indicated at each branch nodes, echinoids (*Lytechinus variegatus*) were rooted to be outgroup species.

## Data Availability

The genome sequence data that support the findings of this study are openly available in GenBank of NCBI at (https://www.ncbi.nlm.nih.gov/) under the accession no. MW248463. The associated BioProject, SRA, and Bio-Sample numbers are PRJNA734962, SRR14725728, and SAMN19551203, respectively.
